# *In vitro* Models of the Human Blood–Brain Barrier and the Impact of Efflux Transporters on Neurological Disorders: The Work of Cioni et al. (2012)

**DOI:** 10.3389/fpsyt.2014.00128

**Published:** 2014-09-16

**Authors:** Silke Vogelgesang, Gabriele Jedlitschky

**Affiliations:** ^1^Department of Neuropathology, Institute of Pathology, University of Greifswald, Greifswald, Germany; ^2^Department of Pharmacology, Center of Drug Absorption and Transport (C_DAT), University of Greifswald, Greifswald, Germany

**Keywords:** blood–brain barrier, P-glycoprotein, St. John’s wort, Alzheimer’s disease

The blood–brain barrier (BBB) plays a fundamental role in the integrity of brain homeostasis. Acute disruption and also even slight shifting of equilibrium of structural elements or transport processes might lead to substantial consequences, resulting in neurological disorders (1).

There is a fundamental need for the development and improvement of *in vitro* models of the BBB in order to understand the biology of these important regulatory tissues, and also to target the BBB in new approaches to the treatment of neurological diseases such as Alzheimer’s disease (AD), stroke, and Parkinson’s disease (2). Capillaries were first successfully isolated from the rat brain in 1973 (3). Since then, a number of *in vitro* models have been developed that are based on primary endothelial cells isolated from several species, the most widely used being rodent, porcine, or bovine cells (4), or on immortalized human brain endothelial cell lines such as hCMEC/D3 cells (5). These monoculture systems, however, are a rather simplified *in vitro* model in that the interactions of endothelial cells with neighboring cells of the neurovascular unit, namely, astrocytes and pericytes, are important for the development and maintenance of BBB properties (1, 6). A fundamental characteristic of the BBB is the formation of interendothelial tight junctions, which is indicated by high transendothelial electrical resistance (TEER) values and the expression of representative tight junction proteins such as occludin (7). Furthermore, a broad range of membrane transporters are expressed at the BBB. ATP-binding-cassette (ABC) efflux transporters such as P-glycoprotein (P-gp, ABCB1) have been identified as key elements of the BBB that prevent many drugs and neurotoxic substances from entering the brain and that transport toxic metabolites out of the brain (2, 8). Astrocytes are important not only for the development of tight junctions but also for the up-regulation of efflux transporters such as P-gp (9). The most widely used *in vitro* models are based on non-human mammalian cells. However, there are species differences in expression levels, substrate affinities, and the regulation of relevant transporters (10). Especially for investigating BBB alterations in the pathogenesis of neurological diseases, the model should mimic the characteristics of the adult human BBB as closely as possible. On the other hand, the model should be reasonable in terms of cost, efficiency, and technical requirements.

Cioni et al. have developed a unique double compartment-model of the BBB consisting of cerebral endothelial cells isolated from cryopreserved human glial tumors. The endothelial cells are isolated together with astroglial cells from the same specimens. Special emphasis was placed by the authors on the activity of drug efflux transporters such as P-gp and members of the multidrug resistance protein- (MRP, ABCC) family, as well as solute carrier (SLC)-type transporters such as organic anion-transporting polypeptides (OATPs). Cioni et al. demonstrated that this brain endothelium culture system mimics a physiologically relevant situation, and may therefore provide a new tool for studying the effects of biological fluids or modulating substances at the BBB. A major advantage of this system is the use of cryopreserved material, which enables the collection of sufficient human brain endothelial cells for investigation (11).

The role of the ABC transporters in the pathogenesis of neurodegenerative disorders such as AD has been increasingly well-established in recent years. P-gp actively transports the amyloid-β (Aβ) peptides Aβ1–40 and Aβ1–42, which represent two of the most prevalent forms of the peptide in senile plaques and cerebral Aβ angiopathy in the AD brain (12, 13). A decrease in P-gp expression has been found in the brains of non-demented aged humans, and low P-gp expression was associated with an increase in cerebral Aβ load (14, 15). It was therefore proposed that induction of P-gp function could restore the clearance of Aβ peptides from the brain to blood via the BBB (16). Furthermore, animal models of AD have been used to investigate the effects of pharmacological P-gp modulation. Because of its known P-gp-inducing effects, one potential candidate has been St. John’s wort (SJW). Brenn et al. showed that mice receiving SJW extract showed significant reductions of parenchymal Aβ1–40 and 1–42 accumulation, and significant increases in cerebrovascular P-gp expression (17). Furthermore, studies have revealed that specific SJW extracts both attenuate Aβ-induced histopathologic changes and alleviate memory impairments in APP-transgenic mice. Interestingly, these effects are independent of hyperforin in the SJW, suggesting that other components of SJW might be involved in its beneficial effects (18). In any case, these studies show that the induction of ABC transporters such as P-gp may be a novel therapeutic strategy to protect the brain from Aβ accumulation, and thus, could impede the progression of neurologic diseases such as AD.

*In vitro* models such as that described by Cioni and colleagues may help to reveal the underlying mechanisms by which BBB transporters influence pathogenic processes, and thus, could point the way toward more effective therapies in humans.

## Conflict of Interest Statement

The authors declare that the research was conducted in the absence of any commercial or financial relationships that could be construed as a potential conflict of interest.
